# Leptomeningeal disease and tumor dissemination in a murine diffuse intrinsic pontine glioma model: implications for the study of the tumor-cerebrospinal fluid-ependymal microenvironment

**DOI:** 10.1093/noajnl/vdac059

**Published:** 2022-04-26

**Authors:** Shelei Pan, Dezhuang Ye, Yimei Yue, Lihua Yang, Christopher P Pacia, Dakota DeFreitas, Prabagaran Esakky, Sonika Dahiya, David D Limbrick, Joshua B Rubin, Hong Chen, Jennifer M Strahle

**Affiliations:** Department of Neurosurgery, Washington University School of Medicine, Saint Louis, Missouri, USA; Department of Mechanical Engineering and Materials Science, Washington University in St. Louis, Saint Louis, Missouri, USA; Department of Biomedical Engineering, Washington University in St. Louis, Saint Louis, Missouri, USA; Department of Radiation Oncology, Washington University School of Medicine, Saint Louis, Missouri, USA; Department of Pediatrics, Washington University in St. Louis, St Louis, Missouri, USA; Department of Biomedical Engineering, Washington University in St. Louis, Saint Louis, Missouri, USA; Department of Neurosurgery, Washington University School of Medicine, Saint Louis, Missouri, USA; Department of Neurosurgery, Washington University School of Medicine, Saint Louis, Missouri, USA; Department of Pathology and Immunology, Washington University School of Medicine, St. Louis, Missouri, USA; Department of Neurosurgery, Washington University School of Medicine, Saint Louis, Missouri, USA; Department of Pediatrics, Washington University in St. Louis, St Louis, Missouri, USA; Department of Pediatrics, Washington University in St. Louis, St Louis, Missouri, USA; Department of Neuroscience, Washington University in St. Louis, St Louis, Missouri, USA; Department of Mechanical Engineering and Materials Science, Washington University in St. Louis, Saint Louis, Missouri, USA; Department of Biomedical Engineering, Washington University in St. Louis, Saint Louis, Missouri, USA; Department of Radiation Oncology, Washington University School of Medicine, Saint Louis, Missouri, USA; Department of Neurosurgery, Washington University School of Medicine, Saint Louis, Missouri, USA; Department of Pediatrics, Washington University in St. Louis, St Louis, Missouri, USA; Department of Orthopedic Surgery, Washington University School of Medicine, St Louis, Missouri, USA

**Keywords:** cerebrospinal fluid, diffuse intrinsic pontine glioma, hydrocephalus, leptomeninges, subventricular zone

## Abstract

**Background:**

Leptomeningeal disease and hydrocephalus are present in up to 30% of patients with diffuse intrinsic pontine glioma (DIPG), however there are no animal models of cerebrospinal fluid (CSF) dissemination. As the tumor–CSF–ependymal microenvironment may play an important role in tumor pathogenesis, we identified characteristics of the Nestin-tumor virus A (Nestin-Tva) genetically engineered mouse model that make it ideal to study the interaction of tumor cells with the CSF and its associated pathways with implications for the development of treatment approaches to address CSF dissemination in DIPG.

**Methods:**

A Nestin-Tva model of DIPG utilizing the 3 most common DIPG genetic alterations (H3.3K27M, PDGF-B, and p53) was used for this study. All mice underwent MR imaging and a subset underwent histopathologic analysis with H&E and immunostaining.

**Results:**

Tumor dissemination within the CSF pathways (ventricles, leptomeninges) from the subependyma was present in 76% (25/33) of mice, with invasion of the choroid plexus, disruption of the ciliated ependyma and regional subependymal fluid accumulation. Ventricular enlargement consistent with hydrocephalus was present in 94% (31/33). Ventricle volume correlated with region-specific transependymal CSF flow (periventricular T2 signal), localized anterior to the lateral ventricles.

**Conclusions:**

This is the first study to report CSF pathway tumor dissemination associated with subependymal tumor in an animal model of DIPG and is representative of CSF dissemination seen clinically. Understanding the CSF–tumor–ependymal microenvironment has significant implications for treatment of DIPG through targeting mechanisms of tumor spread within the CSF pathways.

**Key Points**

- CSF pathway tumor dissemination is observed in DIPG Nestin-Tva GEMMs.
- We observe ependymal and subependymal damage and localized transependymal CSF flow.
- This model offers a new avenue for study of tumor–CSF–ependymal interaction in DIPG.

**Importance of the Study**

Preclinical models of diffuse intrinsic pontine glioma (DIPG) have been widely developed for use in researching the initiation, progression, and treatment of DIPG. Early transplantation-based models used stereotactic implantation of glioma cells into the rodent brain, which were followed by the development of human xenograft models, and later genetically engineered models. The development of these models has largely focused on focal location of tumor in the brainstem and the genetic origin of the tumor. However, as there are currently no curative treatments for DIPG, it is important to consider the cerebrospinal fluid (CSF)–tumor–ependymal microenvironment as a mechanism for tumor pathogenesis. This study is the first to report widespread CSF pathway disease in a DIPG animal model with associated hydrocephalus, ependymal injury, and ependymal/subependymal tumor infiltration. These findings have important implications for DIPG treatment approaches targeting CSF dissemination.

The spread of tumor cells to distant areas of the brain, spine, or meninges is seen in about 20% of DIPG patients,^1–3^ yet there are no published reports on the study of cerebrospinal fluid (CSF) dissemination of tumor, associated hydrocephalus or ependymal injury. Although there have been limited studies of DIPG dissemination through the CSF pathways, recent histopathologic and genetic examination of brain autopsy specimens has revealed extensive intracranial dissemination in DIPG cases,^1,4^ and a seminal study of over 1000 international DIPG patients revealed extrapontine extension of the tumor in 92% of short-term survivors and 86% long-term survivors (survival ≥2 years).^5^ Given the aggressiveness of DIPG and lack of curative treatments, it is necessary to understand tumor spread within the CSF pathways.

Approximately 35% of children with DIPG develop hydrocephalus at an average of 5 months after diagnosis.^6^ This may result from tumor dissemination within the CSF pathways as not all cases of hydrocephalus are from intraventricular obstruction.^7^ Potential mechanisms underlying the development of hydrocephalus including interaction of tumor cells with the CSF and associated pathways (leptomeninges, ventricular ependyma) have not yet been studied,^8^ and as a consequence, the origins and development of DIPG tumor spread and associated hydrocephalus are not clear. DIPGs have been found to spread to the stem cell niche of the subventricular zone (SVZ) of the lateral ventricles in humans,^6^ and previous studies have shown predilection for glioma spread to the SVZ.^9^ These tumor–SVZ interactions within the CSF–tumor–ependyma microenvironment represent an avenue for tumor spread that has not yet been studied in animal models of DIPG.

Over the past several decades, preclinical animal models of glioma have been widely developed and used to study the development and treatment of DIPG tumors. Misuraca et al. described the advantages of genetically engineered mouse models (GEMMs) in providing the opportunity to study tumors that arise in their natural microenvironment in immune-proficient mice.^10^ Over time, GEMMs using the replication-competent avian sarcoma-leucosis virus long-terminal repeat with splice acceptor/tumor virus A (RCAS/Tva) system have evolved to closely parallel the clinical disease, allowing detailed study of the biology of the disease in an in vivo setting. As a result, genetic advances and insight into new mechanisms driving DIPG tumorigenesis have been described.^11^ However, the prevalence of leptomeningeal disease and associated CSF pathway pathology and extra-pontine neuraxis disease in these mice has yet to be examined. Given the clinical rates of CSF pathway spread in DIPG, we evaluated CSF–tumor–ependyma interactions in a Nestin-tumor virus A (Nestin-Tva)-based GEMM of DIPG. We report the first in-depth animal model evaluation of leptomeningeal disease, neuraxis disease, intraventricular dissemination following subependymal tumor infiltration, and hydrocephalus in a DIPG model with implications for clinical pathogenesis and novel treatment approaches.

## Methods

Additional details are provided in Supplementary Material. All animal use protocols were approved by our institution’s Animal Care and Use Committee protocol 20180185.

### Animal Model

P53^flox/flox^ homozygous, Nestin-Tva+ GEMMs of DIPG were used for this study. To generate the model, DF-1 chick fibroblast cells (ATCC CRL-12203) were transfected with one of three RCAS plasmids expressing RCAS-H3.3K27M, RCAS-PDGFB, and RCAS-Cre with FuGENE 6 Transfection Reagent (#E2311, Promega, Madison, WI) to generate the three most highly occurring DIPG genetic alterations (H3.3K27M mutation, PDGFB overexpression, p53 loss).^10^ Once confluent, cells were harvested in a 1:1:1 H3.3K27M:PDGFB:Cre ratio in a 30 µl medium and injected intracranially into postnatal day 7 mice at 3 mm deep of lambda.

Three cohorts of mice were created. The first received a 100 000 DF-1 cell injection into the pons. The second received a 50 000 DF-1 cell injection into the lateral ventricles, and the third received a 50 000 DF-1 cell injection into the pons.

### MRI Imaging

Mice were closely monitored for 3–4 weeks after the DF-1 cell injection, before undergoing T1-weighted post-contrast and T2-weighted MRI in the Small-Animal Magnetic Resonance Facility of the Mallinckrodt Institute of Radiology, Washington University to track tumor development. A cohort of mice also underwent post-contrast T1-weighted and T2-weighted MRI at 5 weeks post-DF-1 cell injection.

T1-weighted MRI images were manually segmented on ITK-SNAP software for quantification of the ventricle and tumor volumes.^12^ T2-weighted images were manually segmented for quantification of the periventricular T2-signal volume.

### Histology

Following the MRI imaging, mice were anesthetized, and a cohort of mouse brains containing tumor were collected (Supplementary Material). Formalin-fixed paraffin embedded sections were stained with H&E to assess tumor extent and examine the ependymal and subependymal wall, and immunohistochemistry for beta-IV tubulin to assess ependymal cilia. 1:200 dilution Olig2 (ab109186, Abcam) and 1:100 dilution Ki67 (ab16667, Abcam) were used to confirm high-grade glioma (HGG) characteristics of the tumor, and 1:400 dilution GFAP (ab7260, Abcam), 1:100 dilution CD45 (ab10558, Abcam), 1:200 dilution CD163 (ab182422, Abcam), and 1:500 dilution CD8 (ab209775, Abcam) were used to examine the immune composition.

### Human Specimens

We obtained sections from the pons and fourth ventricle of a human DIPG specimen (age 12 with postmortem interval 0 h. Sections were stained with H&E to assess tumor extent and ependymal wall integrity. (CMC43450040, Cell Marque, CA, USA), (760-2505, Ventana, AZ, USA), (CMC16321040, Cell Marque, CA, USA), and (790-4460, Ventana, AZ, USA) were used to evaluate the immune composition of tumors.

### Statistical Analysis

All analyses were performed using Microsoft Office Excel (Version 16.36) and Prism (Version 9.3.1 (350)). Associations between continuous variables were assessed using linear regression analysis; DIPG tumor volume was compared to ventricle volume and total periventricular T2 signal volume in separate tests, and ventricle volume was compared to total periventricular T2 signal volume. All tests were 2-tailed, and *P* values of less than .05 were considered statistically significant.

## Results

### Tumor in CSF Pathways

**Figure 1.**
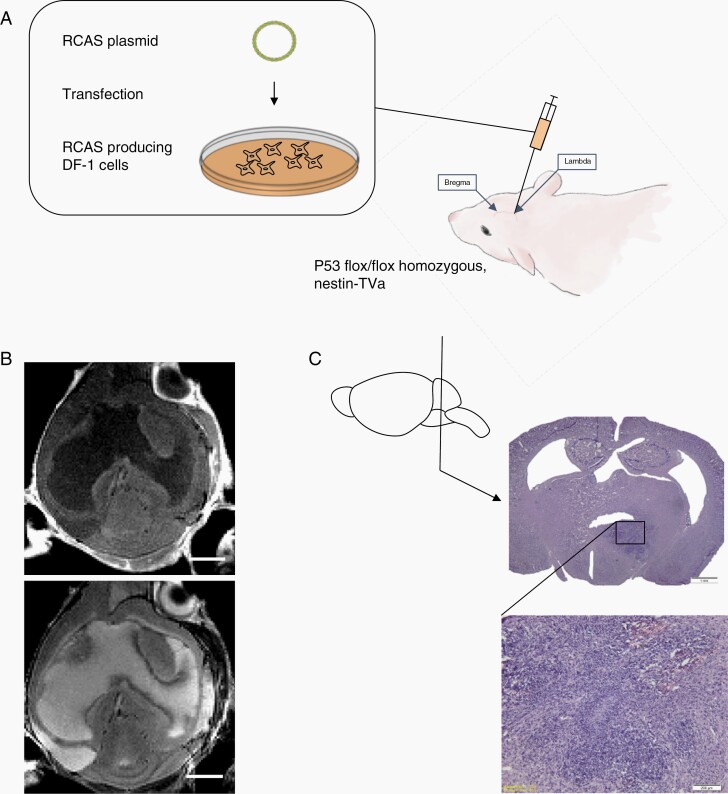
RCAS-based DIPG tumor model. (A) RCAS plasmids are transfected into DF-1 chick embryo cells. These cells produce RCAS viruses and are directly injected into the brainstem of postnatal day 7 mice. (B) Three weeks after DF-1 chick fibroblast injection, mice underwent T1 post-contrast weighted (top) and T2-weighted (bottom) MRI imaging. (C) After MRI, mice were sacrificed, and brains collected. Histology using hematoxylin and eosin (H&E) shows location of tumor. Higher magnification (10×) H&E staining shows tumor growth in the brainstem characterized by patches of basophilic staining with high mitotic activity, pseudopalisading necrosis, and hemorrhage.

**Figure 2.**
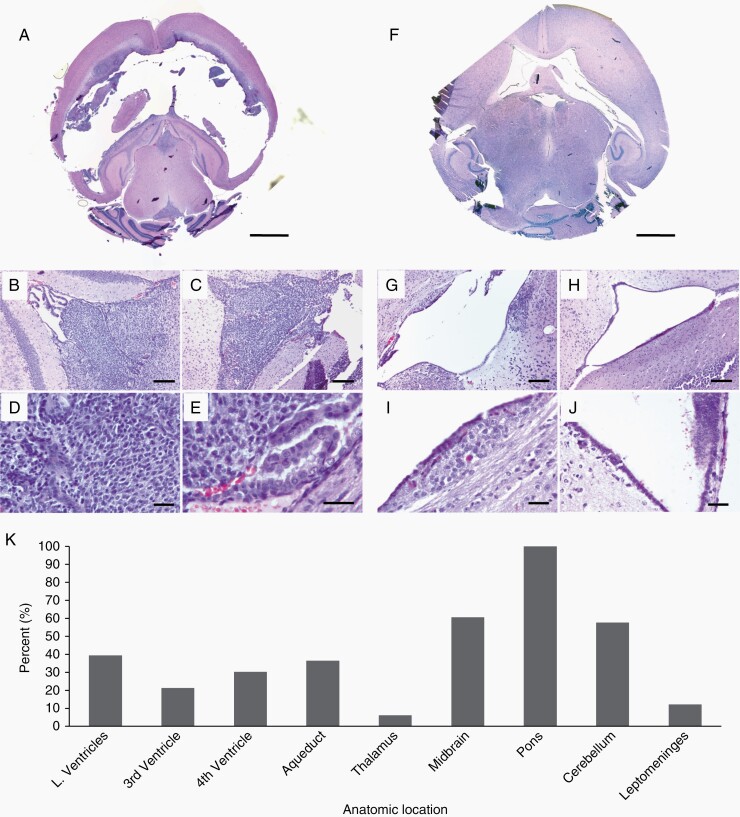
CSF Tumor dissemination in the lateral, third, and fourth ventricles and surrounding brain regions following pontine injection of DF-1 cells. (A) Representative H&E image of severe lateral ventriculomegaly and tumor cells filling the third (B) and fourth (C) ventricles 4 weeks post-intrapontine injection of 100 000 DF-1 cells. Higher magnification images (D, E) show pseudopalisading necrosis and pyknotic cells. (A) 2.5× magnification, scale bar = 5 mm; (B–C) 10× magnification, scale bar = 200 um; (D) 40× magnification, scale bar = 20 um; (E) 63× magnification, scale bar = 20 um. (F) Representative image showing moderate ventriculomegaly with minimal tumor cell presence in the third (G) and fourth (H) ventricles. Higher magnification images (I, J) show subependymal tumor infiltration in the walls of the third ventricle. (F) 2.5× magnification, scale bar = 5 mm; (G–H) 10× magnification, scale bar = 200 um; (I–J) 40× magnification, scale bar = 20 um. (K) Frequency of tumor invasion in different anatomical sites.

When 100 000 DF-1 cells were injected into the brainstem (Figure 1, Supplementary Figure 1) basophilic, mitotic tumor cells were observed along the subependyma and CSF spaces (including the lateral, third and fourth ventricles, cerebral aqueduct, and leptomeninges) (Figure 2). Of the 7 mice with H&E from the 100 000 DF-1 cell pontine injection cohort, 43% (3/7) had tumor throughout the lateral ventricles. Tumor dissemination in the lateral ventricle space was closely associated with subependymal tumor growth, which occurred in 71% (5/7) of the mice in the 100 000 DF-1 cell pontine injection cohort (Table 1). The fourth ventricle was filled with tumor cells in 57% (4/7) (Figure 2B and D), while the third ventricle was filled with tumor cells in 43% (3/7) (Figure 2C and E). Tumor cells were present in the cerebral aqueduct in 100% (7/7) mice, and 57% (4/7) had aqueducts that were completely obstructed by tumor cells (Supplementary Figure 2, Table 1). On MRI, 76% (25/33) were noted to have CSF pathway tumor, indicated by contrast enhancement on post-contrast T1-weighted MRI. Specifically, 12% (4/33) of mice had leptomeningeal contrast enhancement, (Supplementary Figure 3, Table 1) with additional T1 contrast-enhancing tumor in the lateral ventricles (13/33), third ventricle (7/33), fourth ventricle (10/33), cerebral aqueduct (12/33). Tumor was also present in the thalamus (2/33), midbrain (20/33), and cerebellum (19/33). Tumor was present in the pons in 100% (33/33) of the mice (Figure 2K).

**Table 1.** Tumor dissemination in Nestin-Tva GEMM by injection cohort and location

Injection dose and location	Proportion of animals	Olig2 (Presence +/absence −)	Ki67 (Presence +/absence −)	Nestin (Presence +/absence −)
100 000 DF-1 cells, pontine injection				
Pons (primary) tumor	7/7	+	+	+
LV subependymal tumor	6/7	+	+	+
3V subependymal tumor	7/7	+	+	+
Intraventricular tumor	7/7	+	+	+
Leptomeningeal tumor	4/33	N/A	N/A	N/A
50 000 DF-1 cells, pontine injection				
Pons (primary) tumor	6/6	+	+	+
LV subependymal tumor	5/6	+	+	+
3V subependymal tumor	6/6	+	+	+
Intraventricular tumor	5/6	+	+	+
Leptomeningeal tumor	1/6	N/A	N/A	N/A
50 000 DF-1 cells, lateral ventricle injection				
Pons tumor	0/6	−	−	−
LV subependymal tumor	2/6	+	+	+
3V subependymal tumor	3/6	+	+	+
Intraventricular tumor	2/6	+	+	+
Leptomeningeal tumor	0/6	N/A	N/A	N/A

GEMM, genetically engineered mouse model.

**Figure 3.**
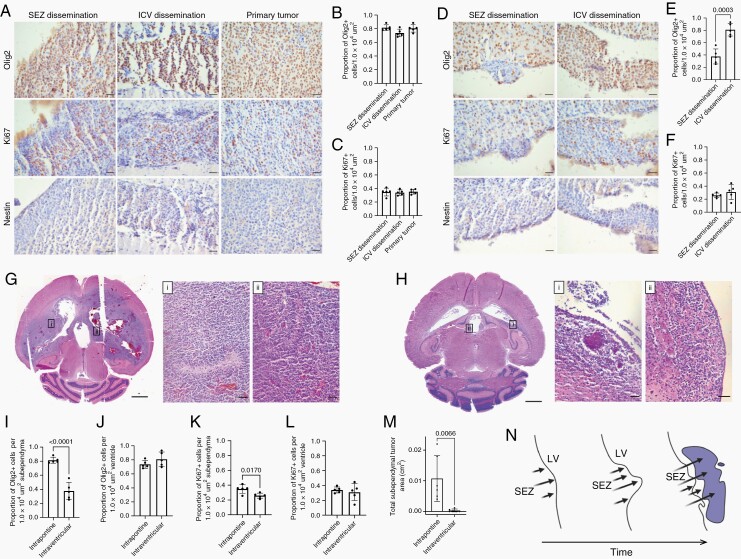
Intraventricular injection of DF-1 cells does not recapitulate the architecture of high grade glioma. (A) Olig2, Ki67, Nestin immunostaining of subependymal (SEZ) dissemination (left column), intraventricular (ICV) dissemination (middle column), and primary (right column) tumor regions 5 weeks post-intrapontine injection of DF-1 cells (50 000 dose). Scale bars = 50 µm. (B–C) Quantification of number of Olig2+ (B) and Ki67+ cells (C) per 1.0 × 10^4^ µm^2^ area corresponding to the conditions in (A). *n* = 6, One-way ANOVA, post-hoc Tukey. (D) Olig2, Ki67, Nestin immunostaining of subependymal (left column) and intraventricular (right column) tumor regions 5 weeks post-lateral ventricle injection of DF-1 cells (50 000 dose). Scale bars = 50 µm. (E–F) Quantification of number of Olig2+ (E) and Ki67+ cells (F) per 1.0 × 10^4^ µm^2^ area corresponding to the conditions in (D). *n* = 6, Unpaired, two tailed *t*-test. (G) Hematoxylin and eosin (H&E) histology of tumor regions 5 weeks post-intrapontine injection of DF-1 cells (50 000 dose). Higher magnification H&E insets of regions indicated in (G) show areas of pseudopalisading necroses from the lateral ventricle subependymal (i) and third ventricle subependymal (ii) tumor. Scale bar = 5 mm, inset scalebars = 50 µm. (H) H&E histology of tumor regions 5 weeks post-lateral ventricle injection of DF-1 cells (50 000 dose). Higher magnification H&E insets of regions indicated in (H) show tumor regions from the lateral ventricle (i) and third ventricle (ii) subependymal tumor. Scale bar = 5 mm, inset scale bars = 50 µm. (I–J) Quantification of number of Olig2+ cells in the subependymal (I) and intraventricular (J) tumor per 1.0 × 10^4^ µm^2^ area in the intrapontine and intraventricular injection cohorts (50 000 dose). *n* = 6, unpaired, two tailed *t*-test. (K–L) Quantification of number of Ki67+ cells in the subependymal (K) and intraventricular (L) tumor per 1.0 × 10^4^ µm^2^ area in the intrapontine and intraventricular cohorts (50 000 dose). *n* = 6, unpaired, two-tailed *t*-test. (M) Quantification of total subependymal tumor area in the intrapontine and intraventricular injection cohorts (50 000 dose). *n* = 6, unpaired, two-tailed *t*-test. (N) Schematic representation of lateral ventricle subependymal tumor growth into the lateral ventricle in the intrapontine injection cohorts over time. LV, lateral ventricle; SEZ, subependymal zone.

To experimentally rule out CSF contamination from the DF-1 cell injection due to technical error as a factor in our observations of CSF pathway tumor, we performed intraventricular injection of DF-1 cells and monitored for tumor development. When we injected the same dose of DF-1 cells (100 000) into the lateral ventricles as we injected into the pons, 2 litters of mice (16 total mice) died within 1 week of injection. We subsequently lowered the intraventricular dose to 50 000 DF-1 cells; there were no deaths and only 33% (2/6) had tumor in any of the CSF spaces (lateral ventricles, third ventricle, fourth ventricle, aqueduct, leptomeninges) (Table 1, Figure 3D). To directly compare with pontine injections, we injected 50 000 DF-1 cells into the pons. In contrast to the 50 000 DF-1 cell intraventricular injection cohort, 83% (5/6) of the mice had tumor in the lateral ventricle, third ventricle, fourth ventricle, or cerebral aqueduct (Figure 3A, Table 1), while one mouse developed leptomeningeal tumor. Overall, there was less tumor in the ventricular system of the intraventricular cohort compared to the intrapontine cohort 5 weeks post-DF-1 cell injection.

### Ependymal Damage and Tumor in the Subependymal and Subventricular Zones

**Figure 4.**
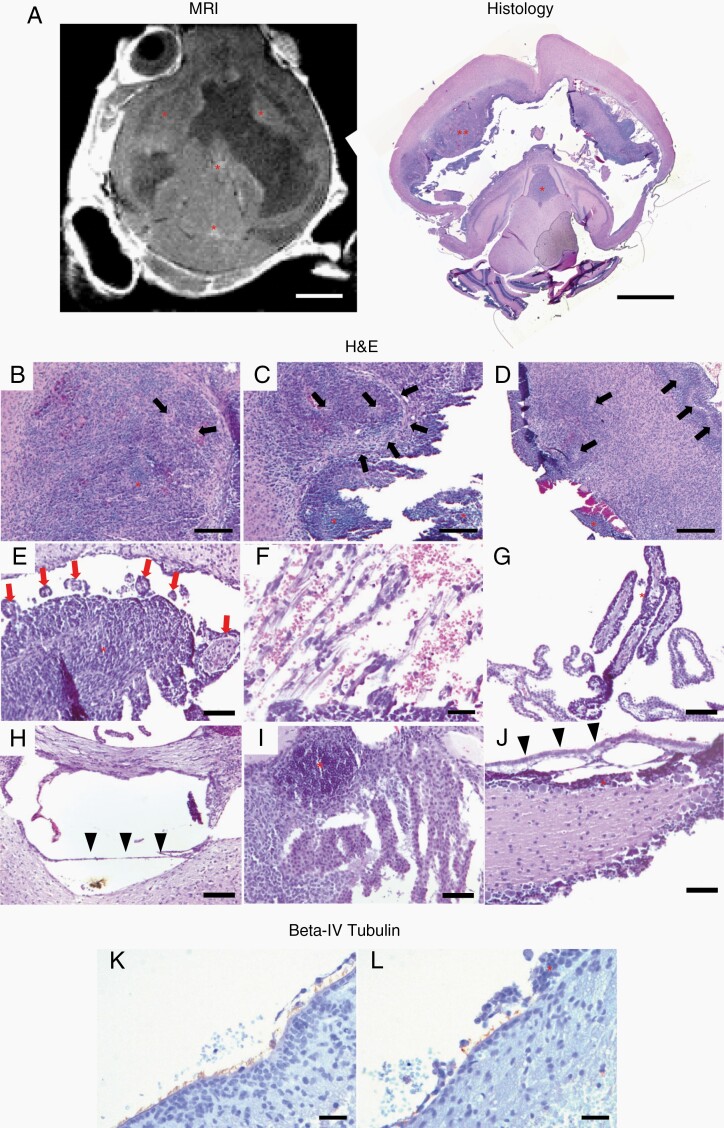
Ependymal, subependymal and choroid plexus response to tumor infiltration following pontine injection of DF-1 cells. Representative H&E (A–J) and beta-IV Tubulin IHC (immunohistochemistry) (K–L) images 4 weeks post-intrapontine injection of 100 000 DF-1 cells. (A) Axial MRI image and photomicrograph showing tumor in the subependyma of the lateral ventricles and within the third and fourth ventricles (red asterisks). 2.5× magnification; scale bar = 5 mm. (B–D) Photomicrograph of the subependyma of the lateral ventricle showing histologic characteristics of high-grade glioma including pseudopalisading necrosis (black arrows) and intratumoral hemorrhage. Red asterisks indicate tumor. (B–D) 10× magnification; scale bars = 200 µm. (E) Photomicrograph of the lateral ventricle subependyma showing granular ependymitis secondary to tumor invasion (red arrows). 20× magnification; scale bars = 50 µm. (F) Photomicrographs showing separation of the ependyma from underlying tissue secondary to subependymal growth of the tumor. Resulting hemorrhage is also present. 40× magnification; scale bars = 20 µm. (G) Tumor dissemination within the epithelium of the choroid plexus. Red asterisk indicates tumor around the surface of the choroid plexus. 20× magnification; scale bars = 50 µm. (H–I) Disruption along the floor of the third ventricle secondary to hydrocephalus and tumor growth. Separation of the third ventricle ependyma (black arrowheads) and tumor interaction with the third ventricle choroid plexus (red asterisk). (H–I) 20× magnification; scale bars = 50 µm. (J) Separation of the ependyma along the fourth ventricle floor. Black arrowheads indicate ependyma, red asterisk indicates tumor. 20× magnification; scale bars = 50 µm. (K–L) Photomicrographs showing cilia lining a section of undisturbed ependyma in the lateral ventricle (K) compared with cilia loss in a section with overlying tumor (L). Tumor is indicated with a red asterisk. (K–L) 40× magnification; scale bars = 20 µm.

Mechanical disruption of the ependyma was present in all seven mice with histopathologic analysis from the 100 000 DF-1 cell pontine injection cohort. On H&E, regions of subependymal tumor were characterized by pseudopalisading necrosis, increased mitotic activity, and areas of hemorrhage. Tumor cells were present from the superior SVZ to the subependymal zone in 43% (3/7) mice (Figure 4A–D), and resulted in separation of the ependyma from the underlying parenchyma (Figures 3A, D and 4B–D). The precise neuroanatomical location not be determined, as the extent of the tumor and the accompanying hydrocephalus grossly distorted the anatomy.

In the frontal horn of the lateral ventricles, tumor cells formed proliferative nodules that expanded outward from the ependyma and subependyma of the ventricle walls into the CSF spaces. Granular ependymitis (round, inflammatory ependymal projections from the wall of the lateral ventricle) (Figure 4E) and filamentous processes with hemorrhage (Figure 4F) were also present.

Additionally, tumor cells surrounded and invaded the choroid plexus of the lateral and third ventricles in several animals (Figure 4G). Cilia was present on the apical surfaces of ependymal cells in regions of undisturbed ependyma, but was only sparsely present in ependymal cells that had underlying or invading tumor growths (Figure 4K and L). This could also be seen in the third (Figure 4H) and fourth (Figure 4J) ventricles.

After intraventricular injection of 50 000 DF-1 cells, there was decreased subependymal tumor infiltration compared to pontine injection of 50 000 DF-1 cells. Thirty-three percent (2/6) of mice with intraventricular injection had lateral ventricle subependymal tumor and 50% (3/6) had third ventricle subependymal tumors (Table 1). In contrast, 83% (5/6) of mice with intrapontine injection had lateral ventricle subependymal tumor, and 100% (6/6) had third ventricle subependymal tumor. Additionally, there was a significant decrease in total subependymal tumor area after 50 000 DF-1 cell intraventricular compared to the 50 000 DF-1 cell pontine injection cohort (Figure 3M). *n* = 6, unpaired, 2-tailed *t*-test.

To confirm that the subependymal and intraventricular tumor recapitulates the biology of HGG, we performed Olig2 and Ki67 immunohistochemistry (Figure 3). In the 50 000 DF-1 cell pontine cohort, there were no differences in Olig2 and Ki67 positivity rate between the subependymal and intraventriclar tumor and primary tumor (Figure 3B and C). *n* = 6, 1-way ANOVA, post hoc Tukey. In contrast, in the 50 000 DF-1 cell intraventricular cohort there were increased Olig2 positivity rates in the ventricle tumor compared to the subependymal tumor (Figure 3E), but no difference in the Ki67 rate (Figure 3F). *n* = 6, unpaired, 2-tailed *t*-test. Overall, there was significantly decreased in Olig2 and Ki67 positivity in subependymal tumor in the intraventricular injection cohort, compared to intrapontine (Figure 3I). *n* = 6, unpaired, 2-tailed *t*-test. There were pseudopalisading necroses in the subependymal tumor of the intrapontine cohort, but not the intraventricular cohort. The subependymal tumor in the intraventricular cohort did not appear to recapitulate the biology of HGG.

### Ventriculomegaly and Hydrocephalus

**Figure 5.**
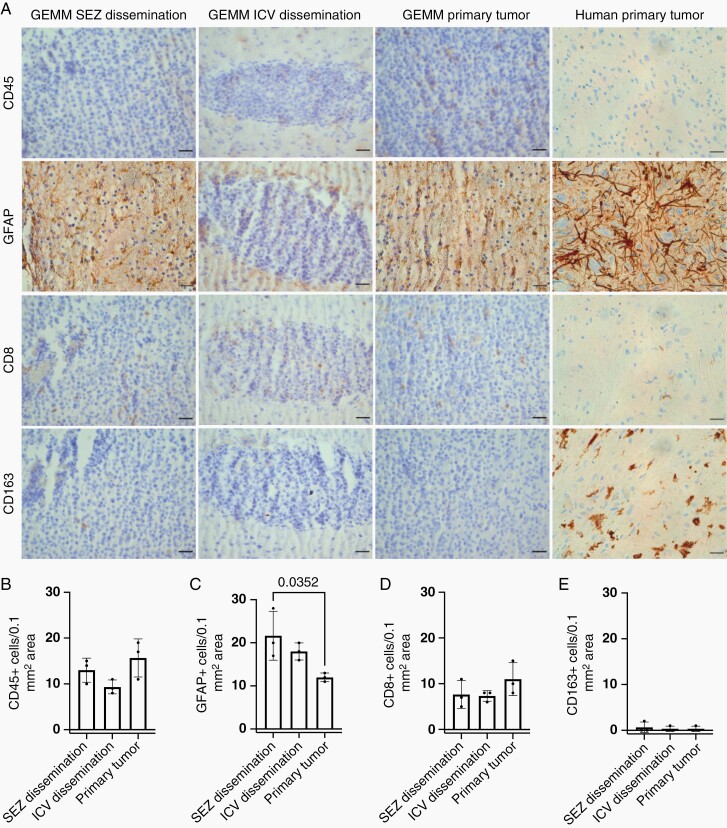
Immune cell profile of GEMM and human DIPG specimen (A) CD45, GFAP, CD8, CD163 immunostaining of subependymal (SEZ) dissemination (far left column), intraventricular (ICV) dissemination (middle left column), and primary (middle right column) tumor regions in the 50 000 DF-1 cell pontine injection cohort of the DIPG Nestin-Tva genetically engineered mouse model (GEMM) and human DIPG legacy specimen (far right column). Scale bars = 25 µm. (B–E) Number of CD45+ (B), GFAP+ (C), CD8+ (D), and CD163+ (E) cells per 0.1 mm^2^ area in the SEZ dissemination, ICV dissemination, and primary tumor areas of the GEMM. Significant differences in the number of GFAP+ cells were observed in the SEZ and primary tumor (*n* = 3 rodents per group, one-way ANOVA with post-hoc Tukey), however no other differences were observed.

Three-to-four weeks post-intrapontine DF-1 cell injection (100 000 dose), mice displayed ventriculomegaly accompanied by doming of the skull, consistent with hydrocephalus (Supplementary Figure 4A). Total ventricle volume ranged from 0.72 to 103.50 mm^3^, and hydrocephalus, defined as ventricle volumes 3 mm^3^ and larger, was present in 94% (31/33) mice from the 100 000 DF-1 cell intrapontine cohort. Tumor volume ranged from 0.136 to 16.03 mm^3^, and was associated with ventricle volume (Supplementary Figure 4D). Qualitatively, in mice with severe ventricular enlargement, brain tissue was reduced to a ring of uneven thickness surrounding the CSF-filled ventricle with an absence of brain tissue between the right and left lateral ventricles in some mice. Discontinuities in the ring of brain tissue were occasionally present, allowing communication between the ventricles and the extra-axial spaces (Figure 5A and C).

### Region-Specific Transependymal CSF Movement

In 88% (29/33) of mice from the 100 000 DF-1 cell intrapontine cohort, the periventricular brain tissue of the lateral ventricles was characterized by hyperintense T2 signal consistent with periventricular fluid secondary to transependymal CSF movement. These areas correspond with hypointense periventricular areas observed on T1 post-contrast images (Supplementary Figure 5A and B) and myelin pallor and edema on H&E (Supplementary Figure 5C and D). Volume of hyperintense T2 signal ranged from 0 to 5.231 mm^3^ and significantly correlated with ventricle volume (Supplementary Figure 5E). The periventricular T2 signal was localized anterior to the lateral ventricles in 100% (29/29) of mice with periventricular T2 signal. There was additional diffuse T2 signal posterior to the lateral ventricles in 21% (6/29) of mice and 31% (9/29) of mice had anterolateral signal. The mice with diffuse periventricular T2 signal that extended into the posterior and lateral regions had larger ventricles than the mice with signal that was localized at the anterior regions only (Supplementary Figure 6). *n* = 13, unpaired, 2-tailed *t*-test. Little to no signal was observed medial to the lateral ventricles or around the third and fourth ventricles.

### Subependymal Spread in Human DIPG

Tumor cells characterized by mitoses and pyknosis could be seen along the floor of the 4V and the underlying brain tissue (Supplementary Figure 8). These mitoses were in multiple locations along the subependyma of the fourth ventricular floor and resulted in protrusion of the ependymal cells toward the ventricle (Supplementary Figure 8).

Furthermore, one of the major advantages of the GEMM is the ability to study tumors in an intact immune system, as DIPG tumors do not have an immunosuppressive microenvironment.^13^ To compare the immune composition between the primary, subependymal, and intraventricular tumor in the GEMM to the primary tumor in the human condition, we performed immunostaining (Figure 5). GEMM pontine, subependymal, and intraventricular tumor areas had similar numbers of CD45+, CD8+, and CD163+ cells, and there were no differences in all staining except for GFAP (Figure 5A, B, and D), where there was significantly increased GFAP expression in the subependymal tumor compared to the primary tumor (Figure 5C). Due to the limited samples that could be obtained, we could not quantify differences between the human and GEMM primary tumors however they qualitatively appeared to have similar numbers of CD45+, GFAP+, and CD8+ cells, while the human primary tumor appeared to have more CD163 expression than the GEMM. While tumor could be seen along the floor of the 4V and the underlying pontine brain tissue in the human specimen (Supplementary Figure 8), human DIPG dissemination along the lateral ventricle subependyma and within the lateral ventricles has not been well described, and we were not able to obtain samples to characterize them.

## Discussion

Leptomeningeal spread of tumor cells is seen in up to 30% of human cases of DIPG.^1–3,14^ However, current treatment with radiation therapy is limited to the pons, and research on novel therapies for DIPG is largely focused on focal drug delivery to the pons. We report the occurrence of leptomeningeal spread of tumor cells in 12% of mice in this DIPG model. As leptomeningeal disease has not been extensively described in animal models of DIPG, this Nestin-Tva model represents a biologically relevant system to study DIPG and address CSF dissemination of subependymal tumor spread through understanding the CSF–tumor–ependyma microenvironment and associated hydrocephalus.

Previously, ependymal disruption was thought to occur through direct damage secondary to expansion of the ventricles by hydrocephalus.^15^ However, through more recent understanding of ependymal biology in the setting of DIPG, there are likely more complex interactions between tumor cells and the ependymal and subependymal regions.^16–18^ In mice with the most pronounced ventricular enlargement and leptomeningeal tumor spread in the pontine injection cohorts, tumor cell proliferation was also present in subventricular and subependymal regions and caused the ependyma to protrude into the ventricles. These histological observations were similar to those in a 2014 autopsy series of 16 DIPG patients showing ventricular and subventricular disease in 10/16 of the patients.^19^ In this model tumor cells infiltrated other CSF contacting surfaces, including the apical surfaces of the choroid plexus. The tumor cells did not invade into the vascular or connective tissue body of the choroid plexus, suggesting the disease was localized to the epithelial cells of the choroid plexus.

The leptomeningeal disease and damage to the ependyma and subependymal tissue may be directly associated with disruption of the SVZ, as the tumor cells observed in the SVZ of our mice were continuous with tumor cells in subependymal and ependymal regions. The SVZ of the lateral ventricles is a source of neural stem cells during the early postnatal period,^20^ and alterations in the SVZ may affect the downstream function of these neuronal stem cells with implications for the development of hydrocephalus.^21–23^ It has been previously suggested that pediatric HGGs, including DIPG, seek the SVZ stem cell niche as a sanctuary site for growth or that SVZ neuronal stem cells harbor driver mutations and contribute to tumorigenesis.^24,25^ As DIPG cells are developmentally similar to cell populations within the SVZ, there may be common trophic signals resulting in their spread to this region.

Similar to autopsy data showing subventricular tumor spread in the lateral and third ventricles,^9,19^ we observed growth adjacent to the fourth ventricle in our patient, indicating the potential for widespread subependymal spread. Owing to the size of the human lateral ventricles and pontine nature of DIPG, it was not possible to characterize the tumor interaction with the lateral and third ventricles in our patient. These observations suggest, however, that the importance of CSF pathways and subependymal and ependymal structures over the course of DIPG tumor growth are conserved between humans and this mouse model. We speculate that tumor growth and its interaction with the ependymal and subependymal tissue, including the SVZ, may alter the microenvironment that the ependymal cells reside in and worsen the degree of ventricular expansion beyond the purely obstructive effects of the DIPG tumor. An alternative hypothesis may be that hydrocephalus opens the subependymal and subventricular zones to tumor invasion through ependymal injury, however this is unlikely to be the observed mechanism in this GEMM as we observed tumor extension outward from the subependymal into the ventricles.

In 2016, Misuraca et al. reported the onset of hydrocephalus in a similar model of DIPG using Pax3-Tv-a mice following DF-1 cell injection into the cerebral cortex, however hydrocephalus has not been studied in detail in pontine DIPG mouse models.^26^ Hydrocephalus in our model was defined by ventricle size >3 mm^3^ on MR imaging 3–4 weeks after DF-1 cell injection in the pons, however almost all mice had an increase in ventricular size. In addition, we found that larger tumor volume was associated with larger ventricles. This is not surprising as the majority of mice with hydrocephalus had evidence of an obstructive component that reflected blockage or damage in other areas of the ventricular system along the third and fourth ventricles. Interestingly, in parallel with the small population of 5 mice with small tumors and large ventricles observed in our study, a recent clinical study reported that the development of hydrocephalus was more likely to be associated with smaller tumors at diagnosis.^6^ A potential explanation for this inverse relationship between tumor size and hydrocephalus may be concurrent leptomeningeal disease contributing to ventricular enlargement outside of an obstruction within the ventricular system.

Recent experimental and clinical findings reveal that in addition to bulk,^27^ glymphatic,^28^ and lymphatic routes,^29,30^ CSF may flow from the ventricles throughout the parenchyma toward the subarachnoid space.^31–33^ This flow is known as transependymal flow, and can be defined as the motion of fluids and/or molecules across the ependymal layer of cells surrounding the ventricles and into the brain parenchyma and adjacent structures.^31^ Transependymal flow or transependymal edema is seen in the periventricular brain by T2 hyperintensities on T2-weighted MRIs,^32^ and has been previously reported in humans.^33^ We observed periventricular T2 signal around the anterior and lateral aspects of the lateral ventricles in most mice. T2 bright signal was associated with areas of lighter staining and more diffuse tissue on the H&E images. Localization of the periventricular signal to predominantly anterior regions of the lateral ventricles has been previously reported,^34^ however it has not been well studied. The significance and relationship of this regional ependymal specification to CSF circulation and fluid movement merits further investigation. There may be distinct molecular characteristics of these ependymal regions that allow for fluid movement across the ventricular surface. Finally, as these anterior regions are adjacent to and overlapping the SVZ, similar mechanisms may be responsible to both fluid movement and subependymal tumor spread to this area.

### Limitations

A primary limitation of this study is the method through which the DF-1 cells were injected. Because the DF-1 cell injection is done by hand, DF-1 cells may have been injected into the fourth ventricle. However, the tumor cells were observed throughout the ventricular system, subependyma, and leptomeninges, suggesting that the tumor was able to spread beyond its location in the fourth ventricle. Our study examined only the Nestin-Tva GEMM of DIPG. Nestin expression is not limited to cells in the brainstem regions, unlike Pax3. Previous studies have been able to generate gliomas in the cerebral cortex parenchyma following virus injection into the cerebral cortex in the Nestin-Tva DIPG rodent model used in this present study, but not the Pax3-Tva model, due to the different locations of cells that express these proteins.^25^ However, hydrocephalus was reported after intracerebral injection in Pax-3 GEMMs, suggesting that the leptomeningeal disease and associated hydrocephalus is not just a consequence of this model’s use of Nestin-Tva expressing cells.

Furthermore, we can make several interpretations from the intraventricular injection cohort data. First, the molecular characteristics of the tumors in the intraventricular injection cohort differed from those in the intrapontine injection cohort, as we observed significantly decreased Olig2 expression and the absence of pseudopalisading necrosis. This indicates that the widespread subependymal tumor dissemination observed in the pontine injections were likely not due to contamination of the ventricular system with DF-1 cells due to technical error.

Second, if the intraventricular and subependymal tumor dissemination observed in the 100 000 pontine injection cohort was due to technical error involving a missed injection of the tumor into the lateral ventricles (as opposed to the pons), we would have likely seen similar rates of mortality as we did in our 100 000 intraventricular injections. In contrast, the 100 000 pontine injection cohort was kept alive for 3–4 weeks before sacrifice, and select litters were followed for up to 5–6 weeks for serial imaging. Therefore, it is unlikely that the 100 000 pontine injection cohort DF-1 cells were injected into the CSF spaces as a result of technical error.

Third, in the 50 000 DF-1 cell intraventricular cohorts, not all animals with subependymal tumor had lateral ventricle tumor, however all animals with lateral ventricle tumor had subependymal tumor. The lower rates of subependymal and ventricle tumor development in the intraventricular cohort suggest that direct injection of the tumor through the CSF spaces is an unlikely mechanism for the disease dissemination observed. Instead, these results suggest a general pattern: that intrapontine DF-1 cells follow subependymal and leptomeningeal spread patterns, with growth in the lateral ventricle and third ventricle subependymal zones that expands into the ventricles and fragments. We did not observe evidence of heavy tumor burden in the fourth ventricle subependyma nor fourth ventricle ependymal disruption, suggesting that the tumor cells observed in the cerebral aqueduct and fourth ventricle likely disseminated within the ventricular system following outgrowth and fragmentation from the subependyma.

Serial MRI imaging was performed at 2 time points post-DF-1 cell injection (3 and 5 weeks) and progressive tumor dissemination was identified (Supplementary Figure 7). A longitudinal study including additional scans at predetermined time points prior to 3 weeks postinjection for each animal would allow more detailed evaluation of tumor progression in this model. In addition, future studies targeting the cellular and molecular mechanisms of CSF and tumor movement across and within the ependyma are needed in order to understand the leptomeningeal disease and hydrocephalus in DIPG.

## Supplementary Material

vdac059_suppl_Supplementary_Materials
