# Supplementary material for: Leptomeningeal disease and tumor dissemination in a murine diffuse intrinsic pontine glioma model: implications for the study of the tumor-cerebrospinal fluid-ependymal microenvironment
Source: Neurooncol Adv. 2022 Apr 26;4(1):vdac059. doi: 10.1093/noajnl/vdac059 (PMC9209751; doi:10.1093/noajnl/vdac059)
Supplement: vdac059_suppl_Supplementary_Materials [file vdac059_suppl_supplementary_materials.zip › vdac059_suppl_Supplementary_Material.docx]

**Supplemental Materials and Methods**

***Animal model.*** All studies were conducted in compliance with the institutional animal care and use committee (IACUC) at Washington University in St. Louis. P53^flox/flox^ homozygous, Nestin-TVa+ genetically engineered mouse models (GEMMs) of DIPG were used for this study. The p53fl/fl homozygous, Nestin-TVa mice and the RCAS expression plasmids encoding Cre, PDGF-B, and H3.3K27M were a kind gift from Dr. Oren Becher. The RCAS-Tva system uses retroviral avian leucosis and sarcoma virus families as gene delivery vectors to incorporate the three most highly recurring DIPG genetic alterations: H3.3K27M mutation, PDGFB signaling overexpression, and p53 loss. Specifically, DF-1 chick fibroblast cells (ATCC CRL-12203) were transfected with one of three individual RCAS plasmids (RCAS-H3.3K27M, RCAS-PDGFB, and RCAS-Cre) with FuGENE 6 Transfection Reagent (#E2311, Promega, Madison, WI). 2.5 μg of DNA were used per T25 flask, with at least 3 serial passages. On the day of injection, confluent cells were harvested in a 1:1:1 H3.3K27M:PDGFB:Cre ratio in a 30 μl medium with 0.6-0.8 million cells in total. Postnatal day 7 pups were taken from the mother and anesthetized with cryoanesthesia before 1.2 ul of cell suspension was injected 3 mm deep into the brainstem at lambda over 2 minutes using a Hamilton (Hamilton Company, Reno, NV) syringe. Following the injection, pups were placed in a 37 °C incubator before being returned to the mother. Animals were checked four hours post-injection and then closely monitored. 38 animals were used in total in the 100,000 pontine DF-1 cell cohort, of which 2 died prior to 3 weeks post-injection and 3 were excluded because of poor image quality of MRI scans. Sixteen animals were used in the 100,000 intraventricular DF-1 cell cohort, all of which died. Eight animals were used in the 50,000 intraventricular DF-1 cell cohort, and seven were used in the 50,000 pontine DF-1 cell cohort, none of which died prior to 3 weeks post-injection. Four animals from the 50,000 pontine DF-1 cell cohort died 5 weeks post-injection.

DF-1 cells were authenticated by amplification of the encoded mRNA using qRT-PCR to confirm expression of the cDNA insert in the RCAS virus. All DF-1 cell transfection protocols were adapted from Ahronian et al.^1^

***MRI imaging.*** Animals underwent initial T1-weighted post-contrast and T2-weighted MR imaging to track tumor development at 3 weeks post-injection. MRIs were obtained using an Agilent/Varian DirectDrive 4.7-T (200-MHz) MRI system in the Small-Animal Magnetic Resonance Facility of the Mallinckrodt Institute of Radiology, Washington University. T1-weighted images were acquired with the following sequence parameters: TR 600.000 ms; TE 11.000 ms; TI 20.000 ms; voxel size 1.25 x 1.25 x 1 mm³, and T2-weighted images were acquired with the following sequence parameters: TR 2000.000 ms; TE 52.000 ms; TI 10.000 ms; voxel size 1.25 x 1.25 x 1 mm³.

T1-weighted post-contrast MR images were manually segmented using ITK-SNAP software for ventricle volume and DIPG tumor volume. To obtain ventricle volume, the lateral, third, and fourth ventricles were delineated on 2D cross-sections in the coronal plane of the T1-weighted post-contrast 3D images. For DIPG tumor volume, the anatomic tumor extent was manually delineated on the basis of T1 post-contrast enhancement on 2D cross-sections in the coronal plane of the same 3D images, with cross reference to the T2 weighted MRI. Total periventricular T2 signal was estimated on coronal sections from T2-weighted MRI using manual segmentation with ITK-SNAP software.

***Histology*.** Mice were anesthetized with 2% isofluorane and perfused with 4% paraformaldehyde, and then decapitated and brains removed. Brains were fixed in 4% paraformaldehyde overnight at 4°C, followed by cryoprotection in sucrose, embedded in paraffin, and sectioned in the coronal plane at 15 µm thickness. Sections were then stained with H&E. Briefly, sections were placed in xylene 2 times for 5 minutes each to remove paraffin, before hydration through descending grades of alcohol (100%, 90%, 70%, 50%, 30%) to DDW. Slides were placed in hematoxylin for 1-2 minutes and running tap water for 5 minutes, before being dehydrated in 70%, 90%, and 100% ethanol. Slides were dipped into xylene 2 times for 2 minutes each and mounted with Permount mounting medium (#SP15-100, Thermo Fisher Scientific, Waltham, MA). Histological evaluation was performed single blinded, by a trained observer without knowledge of the location and treatment. Animals with brain tissue displaying these characteristics were further sub-categorized according to the location of the tumor cells in either the cerebral ventricles or the subarachnoid space. Ependymal damage, if present, was classified as either ependymal or subependymal damage.

The ciliated ependyma was assessed with beta-IV tubulin. Briefly, sections were first soaked in xylene to remove paraffin, before hydration through descending grades of alcohol (100%, 95%, 70%, 50%, 30%) to DDW. Sections were then incubated in a methanol-H2O2 mixture before antigen retrieval using Diva Decloaking solution (Biocare Medical, Pacheco, California). Slides were cooled to room temperature, rinsed with PBS and blocked in 5% normal goat serum, 2.5% BSA, 0.5% TX-100 in PBS for one hour. Sections were incubated in a 1:100 dilution of anti-beta-IV tubulin (#11315, Abcam, Cambridge, MA) in PBS with 1% BSA and 0.5% TX-100 overnight at 4°C, rinsed in PBS 6 times for 5 minutes each, and then incubated with anti-mouse IgG, HRP-linked antibody (Invitrogen A-11032, Carlsbad, California) diluted in PBS with 1% BSA, 0.5% TX-100 for 90 minutes at RT in a 1:2000 dilution. Sections were rinsed in PBS and developed in the dark in Stable Diaminobenzidine (DAB), then rinsed with distilled water (DW) under a running tap. Sections were counterstained with hematoxylin for 7 seconds, rinsed with DW under a running tap and then dehydrated with ascending grades of alcohol (30%, 50%, 70%, 95%, 100%) before being soaked in xylene and mounted with Permount mounting medium (#SP15-100, Thermo Fisher Scientific, Waltham, MA).

1. Zhang G, Chi Y, Du YN. Identification and Characterization of Metastatic Factors by Gene Transfer into the Novel RIP-Tag; RIP-tva Murine Model. *J Vis Exp*. 2017;(128):55890. Published 2017 Oct 16. doi:10.3791/55890
