# Supplementary material for: Leptomeningeal disease and tumor dissemination in a murine diffuse intrinsic pontine glioma model: implications for the study of the tumor-cerebrospinal fluid-ependymal microenvironment
Source: Neurooncol Adv. 2022 Apr 26;4(1):vdac059. doi: 10.1093/noajnl/vdac059 (PMC9209751; doi:10.1093/noajnl/vdac059)
Supplement: vdac059_suppl_Supplementary_Materials [file vdac059_suppl_supplementary_materials.zip › vdac059_suppl_Supplementary_Figure_S1.pptx]

## Slide 1
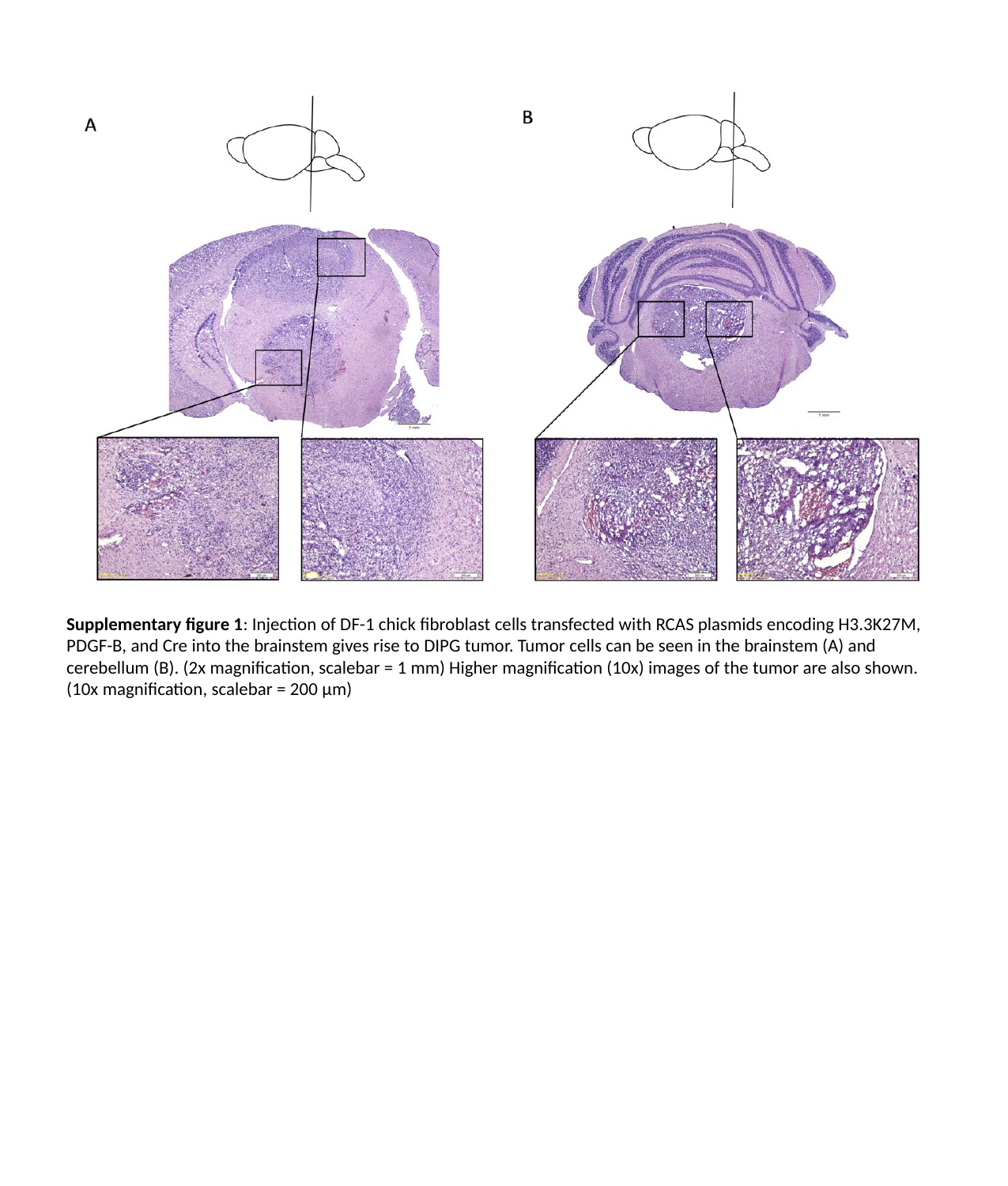

Supplementary figure 1: Injection of DF-1 chick fibroblast cells transfected with RCAS plasmids encoding H3.3K27M, PDGF-B, and Cre into the brainstem gives rise to DIPG tumor. Tumor cells can be seen in the brainstem (A) and cerebellum (B). (2x magnification, scalebar = 1 mm) Higher magnification (10x) images of the tumor are also shown. (10x magnification, scalebar = 200 μm)
