# Supplementary material for: Leptomeningeal disease and tumor dissemination in a murine diffuse intrinsic pontine glioma model: implications for the study of the tumor-cerebrospinal fluid-ependymal microenvironment
Source: Neurooncol Adv. 2022 Apr 26;4(1):vdac059. doi: 10.1093/noajnl/vdac059 (PMC9209751; doi:10.1093/noajnl/vdac059)
Supplement: vdac059_suppl_Supplementary_Materials [file vdac059_suppl_supplementary_materials.zip › vdac059_suppl_Supplementary_Figure_S2.pptx]

## Slide 1
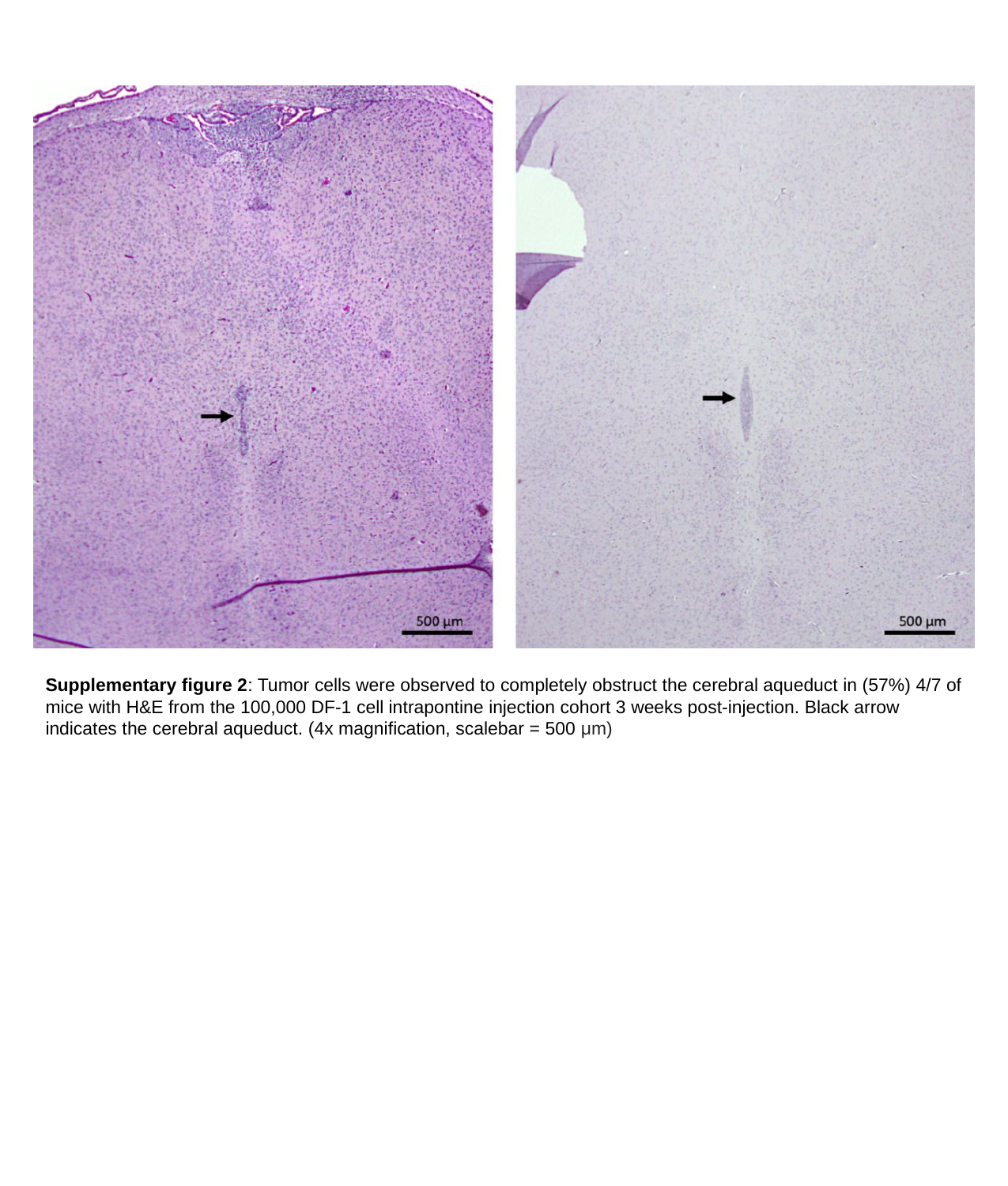

Supplementary figure 2: Tumor cells were observed to completely obstruct the cerebral aqueduct in (57%) 4/7 of mice with H&E from the 100,000 DF-1 cell intrapontine injection cohort 3 weeks post-injection. Black arrow indicates the cerebral aqueduct. (4x magnification, scalebar = 500 μm)
