# Supplementary material for: Leptomeningeal disease and tumor dissemination in a murine diffuse intrinsic pontine glioma model: implications for the study of the tumor-cerebrospinal fluid-ependymal microenvironment
Source: Neurooncol Adv. 2022 Apr 26;4(1):vdac059. doi: 10.1093/noajnl/vdac059 (PMC9209751; doi:10.1093/noajnl/vdac059)
Supplement: vdac059_suppl_Supplementary_Materials [file vdac059_suppl_supplementary_materials.zip › vdac059_suppl_Supplementary_Figure_S3.pptx]

## Slide 1
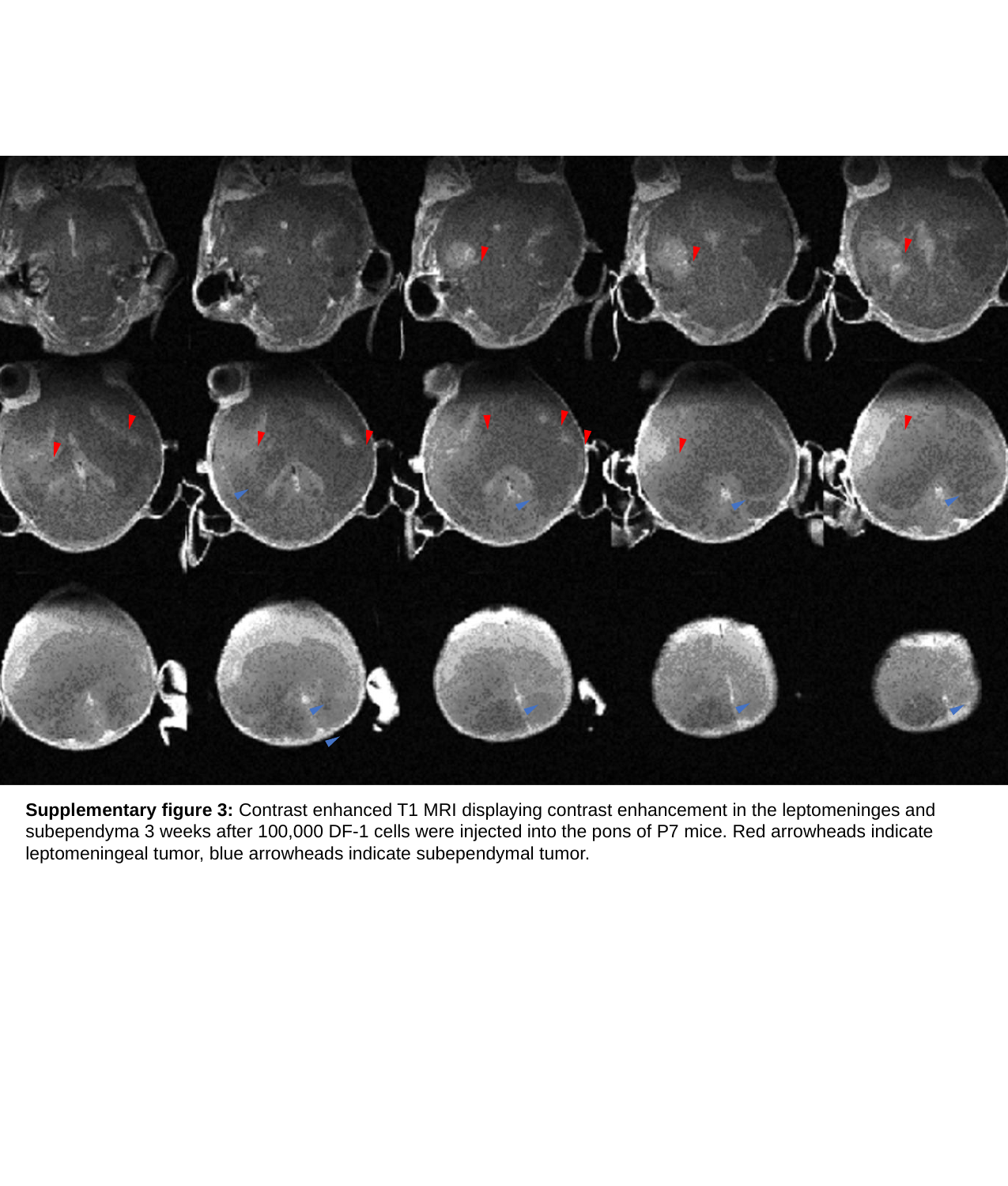

Supplementary figure 3: Contrast enhanced T1 MRI displaying contrast enhancement in the leptomeninges and subependyma 3 weeks after 100,000 DF-1 cells were injected into the pons of P7 mice. Red arrowheads indicate leptomeningeal tumor, blue arrowheads indicate subependymal tumor.
