# Supplementary material for: Leptomeningeal disease and tumor dissemination in a murine diffuse intrinsic pontine glioma model: implications for the study of the tumor-cerebrospinal fluid-ependymal microenvironment
Source: Neurooncol Adv. 2022 Apr 26;4(1):vdac059. doi: 10.1093/noajnl/vdac059 (PMC9209751; doi:10.1093/noajnl/vdac059)
Supplement: vdac059_suppl_Supplementary_Materials [file vdac059_suppl_supplementary_materials.zip › vdac059_suppl_Supplementary_Figure_S4.pptx]

## Slide 1
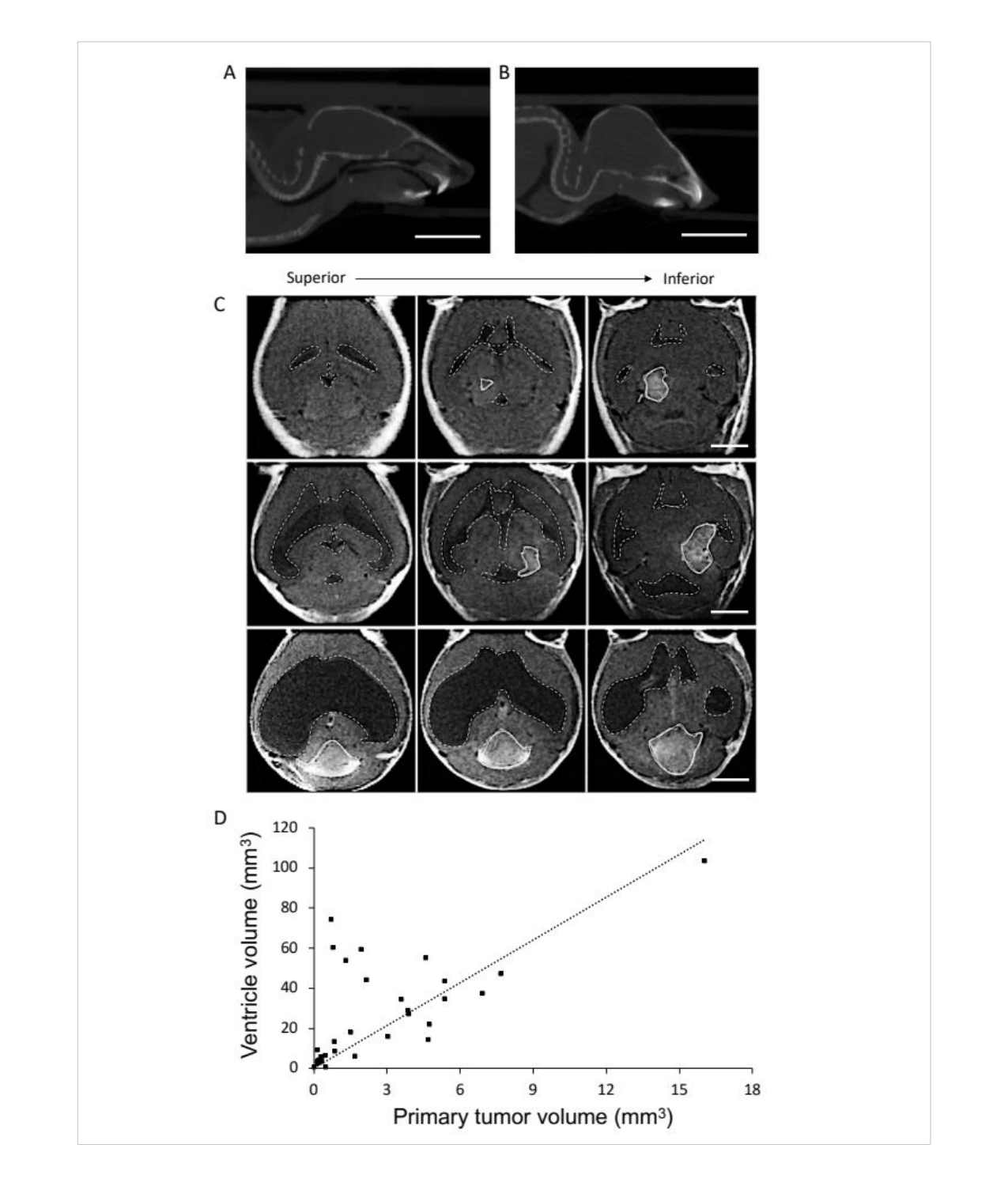

## Slide 2
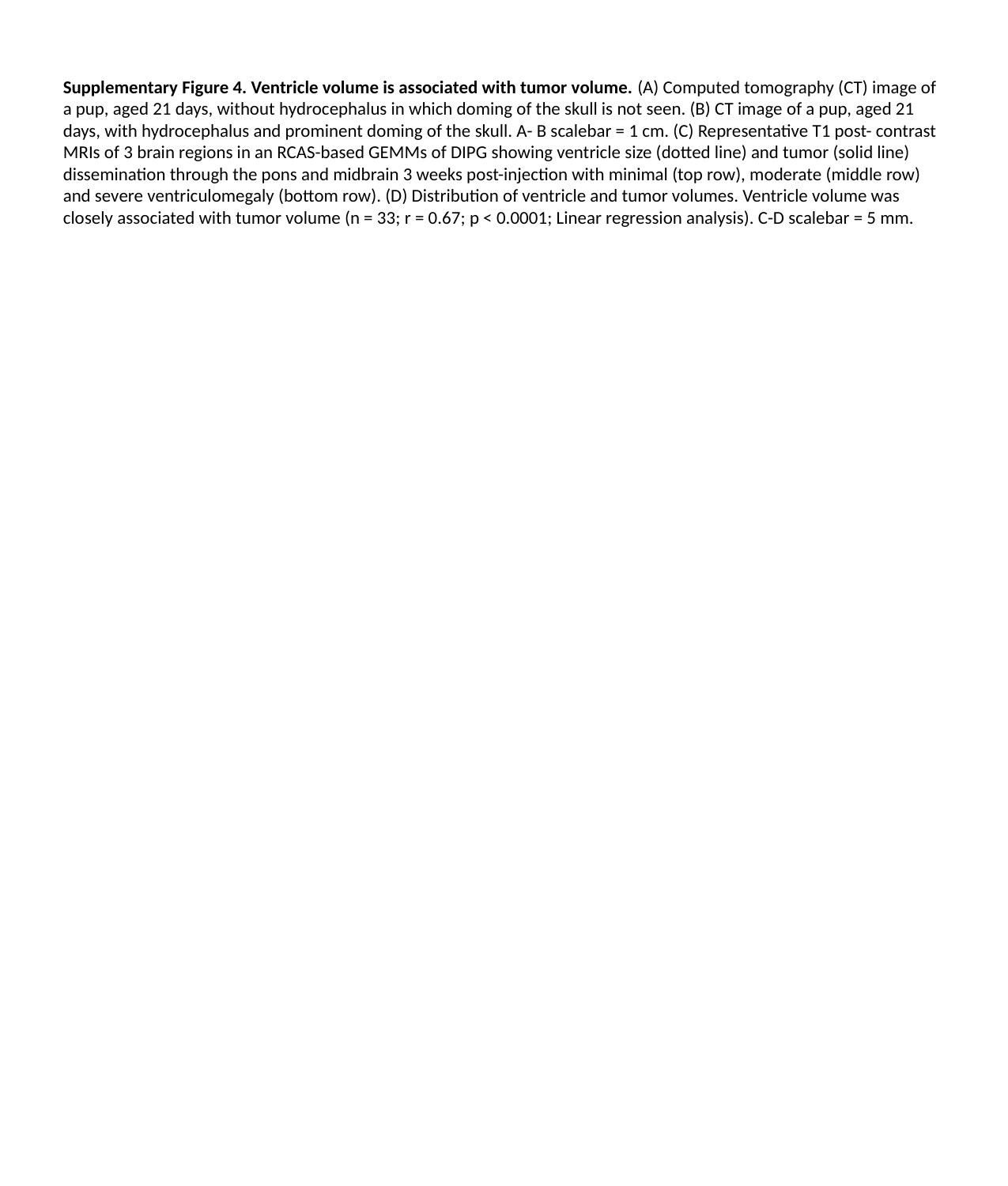

Supplementary Figure 4. Ventricle volume is associated with tumor volume. (A) Computed tomography (CT) image of a pup, aged 21 days, without hydrocephalus in which doming of the skull is not seen. (B) CT image of a pup, aged 21 days, with hydrocephalus and prominent doming of the skull. A- B scalebar = 1 cm. (C) Representative T1 post- contrast MRIs of 3 brain regions in an RCAS-based GEMMs of DIPG showing ventricle size (dotted line) and tumor (solid line) dissemination through the pons and midbrain 3 weeks post-injection with minimal (top row), moderate (middle row) and severe ventriculomegaly (bottom row). (D) Distribution of ventricle and tumor volumes. Ventricle volume was closely associated with tumor volume (n = 33; r = 0.67; p < 0.0001; Linear regression analysis). C-D scalebar = 5 mm.
