# Supplementary material for: Leptomeningeal disease and tumor dissemination in a murine diffuse intrinsic pontine glioma model: implications for the study of the tumor-cerebrospinal fluid-ependymal microenvironment
Source: Neurooncol Adv. 2022 Apr 26;4(1):vdac059. doi: 10.1093/noajnl/vdac059 (PMC9209751; doi:10.1093/noajnl/vdac059)
Supplement: vdac059_suppl_Supplementary_Materials [file vdac059_suppl_supplementary_materials.zip › vdac059_suppl_Supplementary_Figure_S5.pptx]

## Slide 1
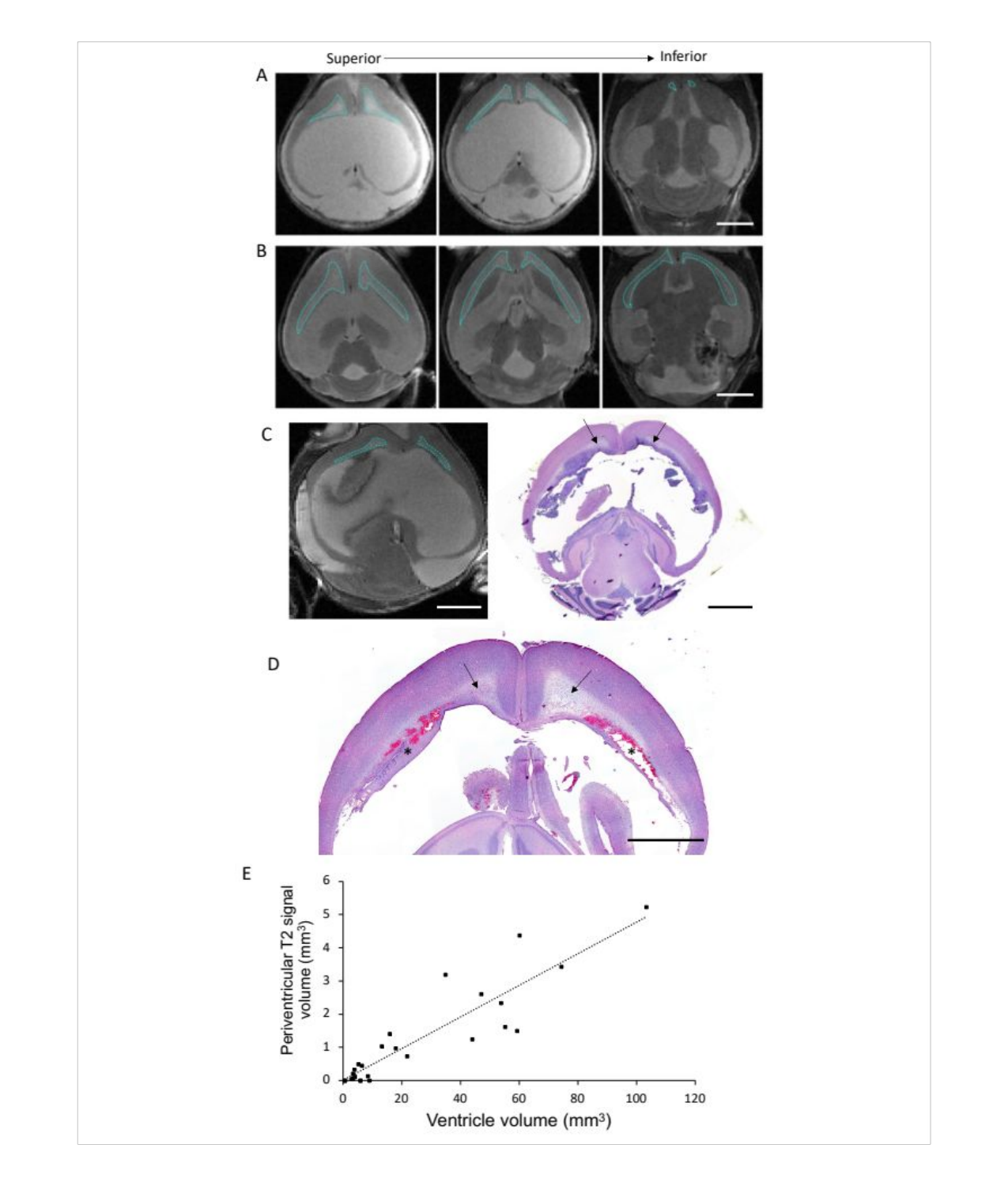

## Slide 2
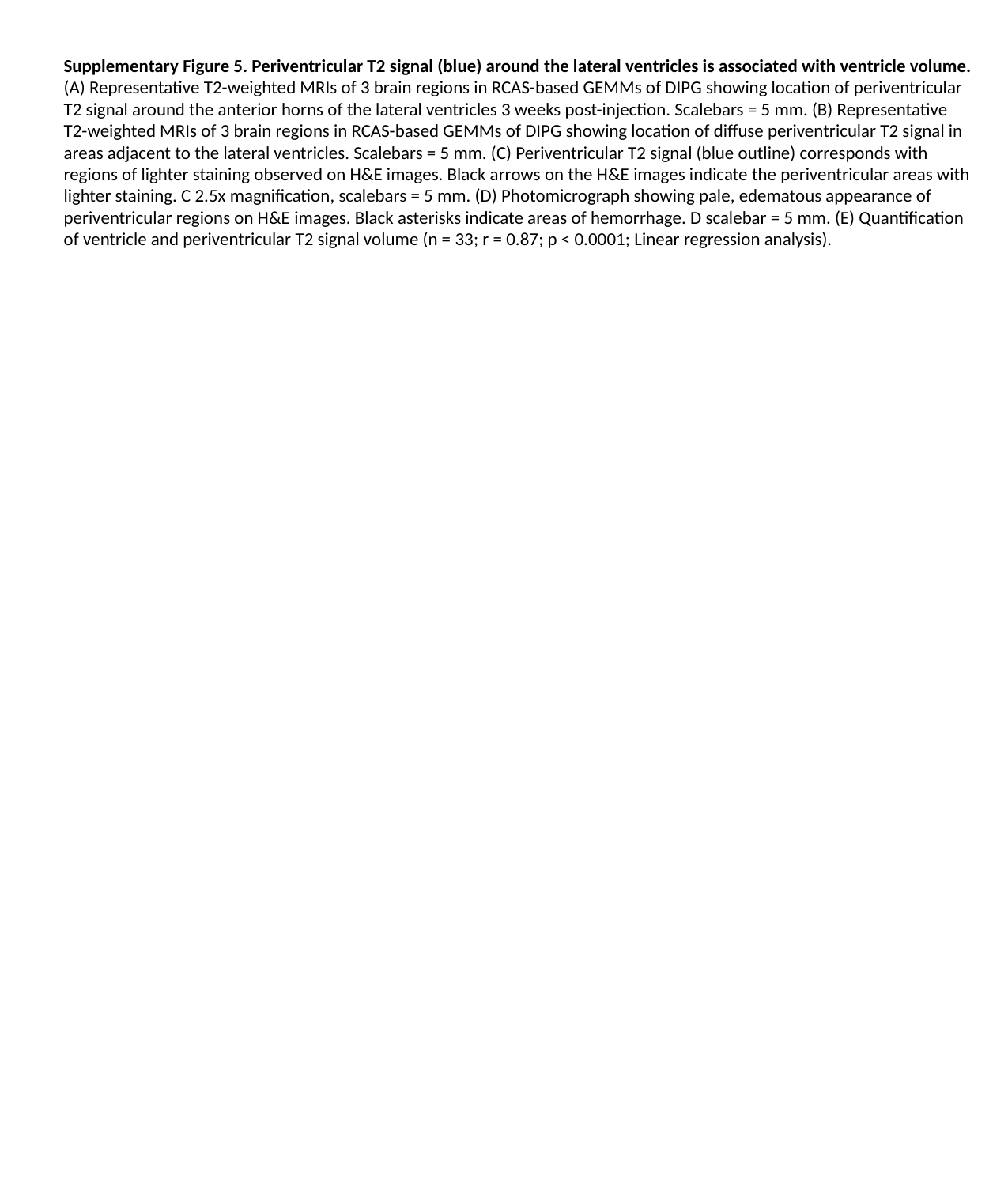

Supplementary Figure 5. Periventricular T2 signal (blue) around the lateral ventricles is associated with ventricle volume. (A) Representative T2-weighted MRIs of 3 brain regions in RCAS-based GEMMs of DIPG showing location of periventricular T2 signal around the anterior horns of the lateral ventricles 3 weeks post-injection. Scalebars = 5 mm. (B) Representative T2-weighted MRIs of 3 brain regions in RCAS-based GEMMs of DIPG showing location of diffuse periventricular T2 signal in areas adjacent to the lateral ventricles. Scalebars = 5 mm. (C) Periventricular T2 signal (blue outline) corresponds with regions of lighter staining observed on H&E images. Black arrows on the H&E images indicate the periventricular areas with lighter staining. C 2.5x magnification, scalebars = 5 mm. (D) Photomicrograph showing pale, edematous appearance of periventricular regions on H&E images. Black asterisks indicate areas of hemorrhage. D scalebar = 5 mm. (E) Quantification of ventricle and periventricular T2 signal volume (n = 33; r = 0.87; p < 0.0001; Linear regression analysis).
