# Supplementary material for: Leptomeningeal disease and tumor dissemination in a murine diffuse intrinsic pontine glioma model: implications for the study of the tumor-cerebrospinal fluid-ependymal microenvironment
Source: Neurooncol Adv. 2022 Apr 26;4(1):vdac059. doi: 10.1093/noajnl/vdac059 (PMC9209751; doi:10.1093/noajnl/vdac059)
Supplement: vdac059_suppl_Supplementary_Materials [file vdac059_suppl_supplementary_materials.zip › vdac059_suppl_Supplementary_Figure_S6.pptx]

## Slide 1
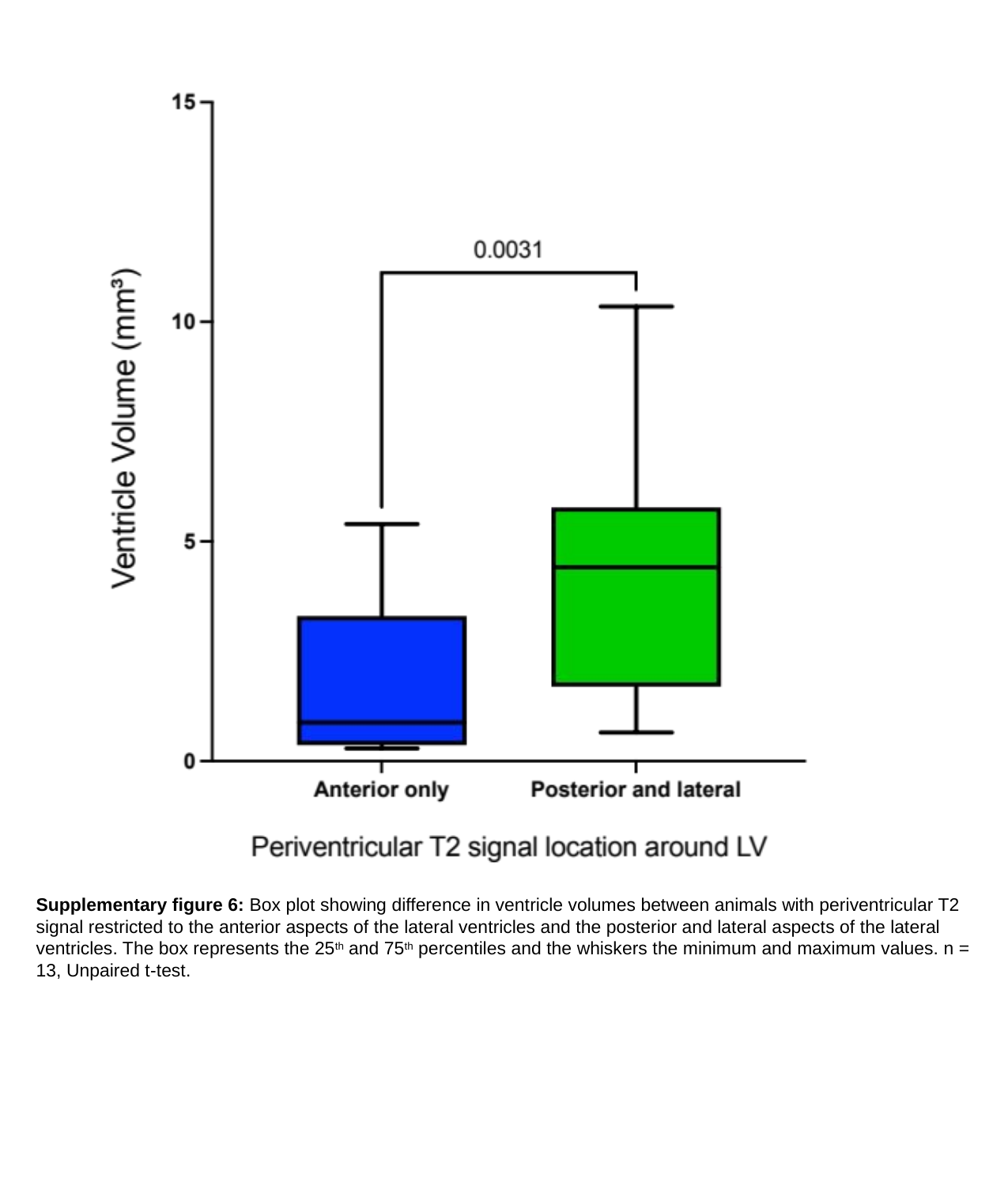

Supplementary figure 6: Box plot showing difference in ventricle volumes between animals with periventricular T2 signal restricted to the anterior aspects of the lateral ventricles and the posterior and lateral aspects of the lateral ventricles. The box represents the 25th and 75th percentiles and the whiskers the minimum and maximum values. n = 13, Unpaired t-test.
