# Supplementary material for: Leptomeningeal disease and tumor dissemination in a murine diffuse intrinsic pontine glioma model: implications for the study of the tumor-cerebrospinal fluid-ependymal microenvironment
Source: Neurooncol Adv. 2022 Apr 26;4(1):vdac059. doi: 10.1093/noajnl/vdac059 (PMC9209751; doi:10.1093/noajnl/vdac059)
Supplement: vdac059_suppl_Supplementary_Materials [file vdac059_suppl_supplementary_materials.zip › vdac059_suppl_Supplementary_Figure_S7.pptx]

## Slide 1
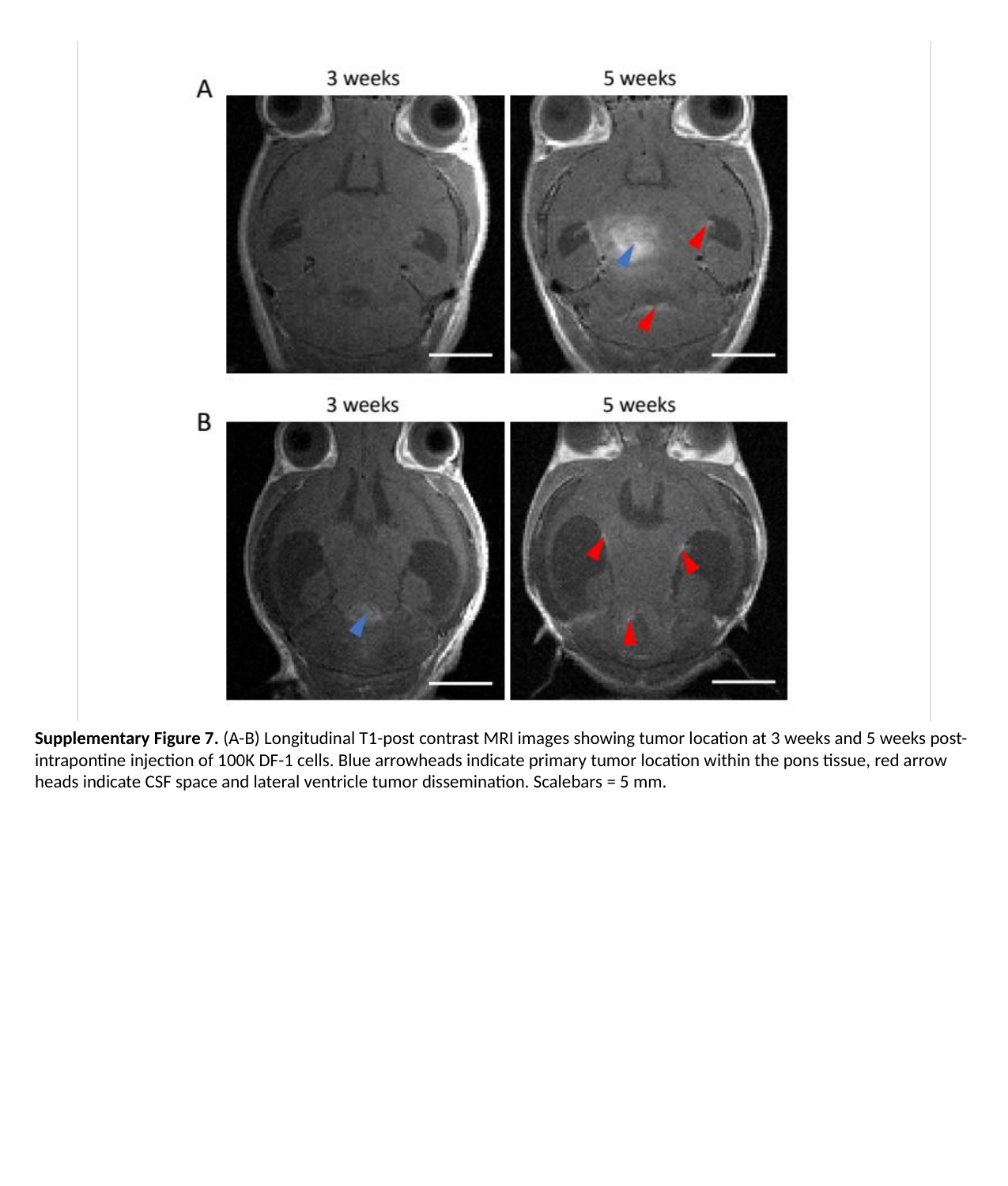

Supplementary Figure 7. (A-B) Longitudinal T1-post contrast MRI images showing tumor location at 3 weeks and 5 weeks post-intrapontine injection of 100K DF-1 cells. Blue arrowheads indicate primary tumor location within the pons tissue, red arrow heads indicate CSF space and lateral ventricle tumor dissemination. Scalebars = 5 mm.
