# Supplementary material for: Leptomeningeal disease and tumor dissemination in a murine diffuse intrinsic pontine glioma model: implications for the study of the tumor-cerebrospinal fluid-ependymal microenvironment
Source: Neurooncol Adv. 2022 Apr 26;4(1):vdac059. doi: 10.1093/noajnl/vdac059 (PMC9209751; doi:10.1093/noajnl/vdac059)
Supplement: vdac059_suppl_Supplementary_Materials [file vdac059_suppl_supplementary_materials.zip › vdac059_suppl_Supplementary_Figure_S8.pptx]

## Slide 1
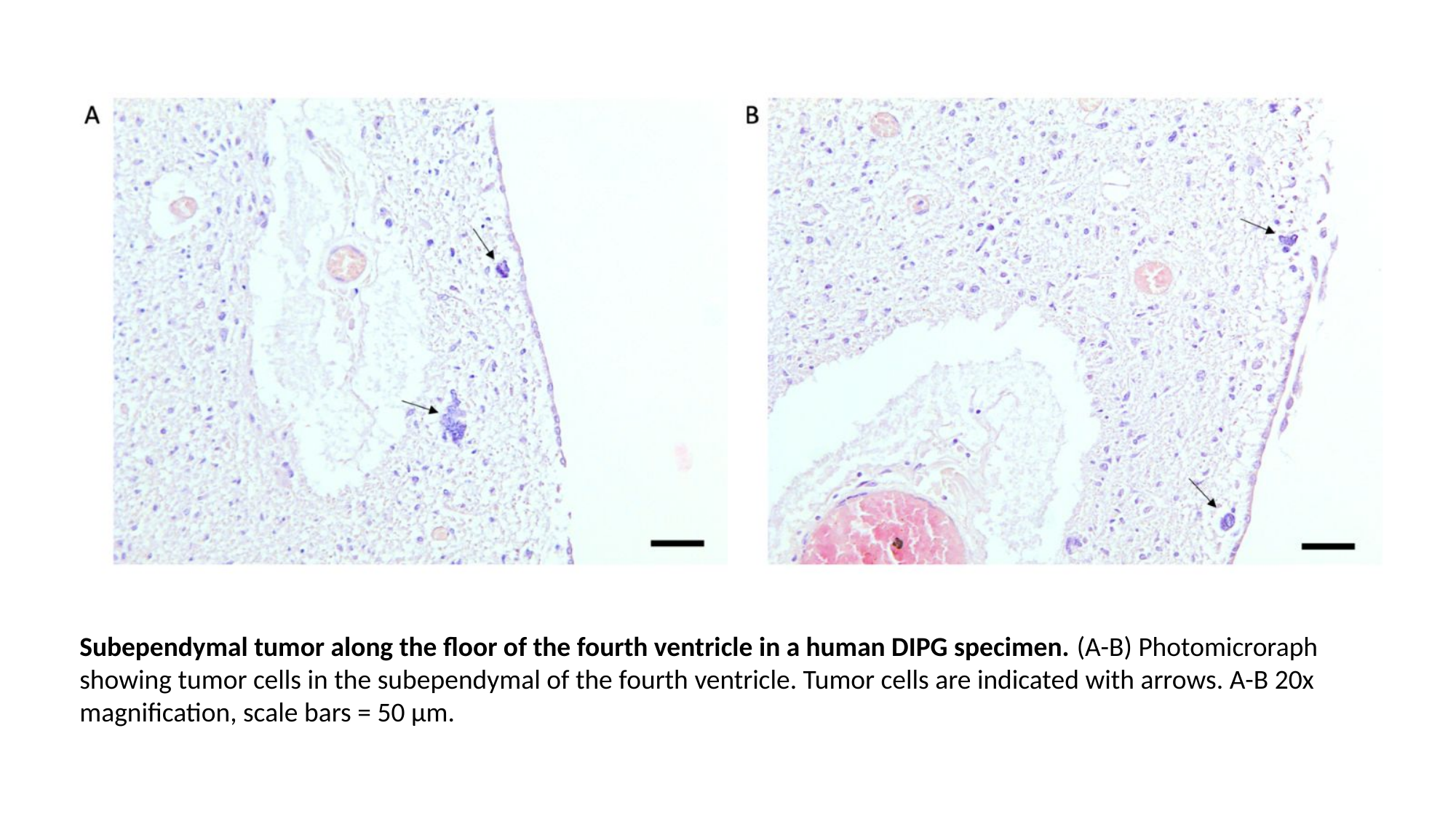

Subependymal tumor along the floor of the fourth ventricle in a human DIPG specimen. (A-B) Photomicroraph showing tumor cells in the subependymal of the fourth ventricle. Tumor cells are indicated with arrows. A-B 20x magnification, scale bars = 50 µm.
